# Efficacy Comparison of Different Acupuncture Treatments for Functional Dyspepsia: A Systematic Review with Network Meta-Analysis

**DOI:** 10.1155/2020/3872919

**Published:** 2020-03-18

**Authors:** Jinhuan Zhang, Yongfeng Liu, Xingxian Huang, Yirong Chen, Liyu Hu, Kai Lan, Haibo Yu

**Affiliations:** ^1^Fourth Clinical Medical School of Guangzhou University of Chinese Medicine, Shenzhen 518033, China; ^2^Shenzhen Traditional Chinese Medicine Hospital, Shenzhen 518033, China

## Abstract

**Background:**

Acupuncture has been found to be an effective treatment for functional dyspepsia (FD). Currently, several types of acupuncture have been developed but it is not clear which type is suitable for FD. Currently, doctors often rely on experience to decide which form of acupuncture to apply. Herein, we employed network meta-analysis (NMA) to compare the effectiveness of various methods of acupuncture in the treatment of functional dyspepsia.

**Methods:**

We searched for randomized controlled trials (RCTs) of acupuncture treatments for functional dyspepsia in seven databases; PubMed, the Cochrane Library, Embase, Wanfang database, China National Knowledge Infrastructure (CNKI) database, Chinese Science and Technique Journals (CQVIP), and Chinese Biomedical Database (CBM) from the date of database inception to October 10, 2019. Cochrane risk of bias tool was used to analyze the risk of bias of the included RCTs. Pairwise meta-analyses were performed with RevMan 5.3 and the network meta-analysis of the included RCTs was performed using the frequentist framework.

**Results:**

A total of 35 studies involving 3301 patients and 10 interventions were eligible for this study. NMA results showed that five types of acupuncture (manual acupuncture, acupoint application, moxibustion, acupoint catgut embedding, and warm acupuncture alone) all were superior to prokinetics (itopride, mosapride, and domperidone) and sham acupuncture in terms of improving the symptoms of functional dyspepsia. Specifically, manual acupuncture and electroacupuncture were more effective in improving the MOS 36 Item Short-Form Health Survey (SF-36) compared to itopride and sham acupuncture, and electroacupuncture was the best among the three acupuncture therapies (acupuncture, electroacupuncture, and acupoint catgut embedding). Moxibustion and manual acupuncture were more effective in improving Nepean Dyspepsia Life Quality Index (NDLQI) compared to itopride, domperidone, and sham acupuncture; moxibustion ranks first among the three acupuncture therapies (acupuncture, electroacupuncture, moxibustion).

**Conclusions:**

These results showed that manual acupuncture alone was the most effective therapy for FD. It should, therefore, be considered as an alternative treatment for FD patients who are unresponsive to prokinetics or intolerant to the adverse effects of prokinetics. We recommend further multiple centers and high-quality RCT studies to confirm the present findings.

## 1. Introduction

Functional dyspepsia (FD) is defined by the Rome IV criteria as recurrent or chronic epigastric symptoms in the absence of organic disease likely to explain them [1]. Dyspepsia is a common disorder diagnosed in clinical practice [2]. Among the types of dyspepsia, the prevalence of FD is nearly 20% [3]. Although FD is not life-threatening, it significantly affects the quality of life and work productivity. It also exerts considerable pressure on health care, societal, and self-costs [4, 5].

Currently, the exact pathophysiology of FD is unknown, but several multiple mechanisms have been proposed [6]. Some of them include motility disorders, gastric hypersensitivity, hypersensitivity to acid and chemical stimuli, immune activation, disorders of the autonomic and enteric nervous systems, and low-grade inflammation [7, 8], all of which affect the phenotypes and severity of FD symptoms. At present, FD treatment is mainly treated with proton pump inhibition, proton pump inhibitors (PPIs), histamine-2 receptor antagonists (H2RAs), anticoke depression drugs, protective agents for gastric mucosa, and antimicrobial agents targeted at *H. Pylori* [9]. However, the effectiveness and safety of these drugs remain controversial [10] and only few among them have well-established efficacy [6]. Recent studies show that domperidone increases the risk of ventricular arrhythmias and prolongation of the QT‐interval [11, 12].

Acupuncture has fewer side effects when used for functional dyspepsia [13]. Research has demonstrated that acupuncture can improve gastrointestinal motility [14]. It has also been observed that electroacupuncture (EA) improves gastric slow waves and accelerates gastric emptying mediated via the autonomic and cholinergic mechanisms [15]. Functional neuroimaging studies have revealed that acupuncture and sham acupuncture have relatively different clinical efficacies and brain responses [16]. Acupuncture improves symptoms by modulating the sensory transduction regions (brainstem and thalamus), visceral modulation regions [17], and nerve activity [18].

In recent years, several systematic reviews [19–22] have shown that different acupuncture therapies effectively improve functional dyspepsia, even better than conventional drugs. So far, many types of acupuncture therapies have been developed, including manual acupuncture [23], warm acupuncture [24], electroacupuncture [25], moxibustion [26], acupoint catgut embedding [27], and acupoint application [28]. Previous systematic reviews [19–22] have only evaluated the efficacy of different acupuncture therapies as a whole in comparison with conventional drugs. In 2017, Ho et al. conducted a systematic review and network meta-analysis on acupuncture and other related therapies for functional dyspepsia. They concluded that a combination of manual acupuncture and clebopride was the most effective approach of attenuating FD symptoms [29]; however, this study conclusion was based on studies published before 2016, and therefore the types of acupuncture included covered were few.

Currently, the choice of acupuncture therapy is subjective and depends on the experience of the acupuncturists. To develop a more standardized approach to guide the clinical application of acupuncture for FD, we employed network meta-analysis to compare the efficacy of acupuncture, warm acupuncture, moxibustion, electroacupuncture, acupoint application, and acupoint catgut embedding in the treatment of functional dyspepsia.

## 2. Methods

See [Supplementary-material supplementary-material-1] in the Supplementary Material for the PRISMA (preferred reporting items for systematic reviews and meta-analyses) extension statement for our network meta-analysis.

### 2.1. Search Strategy

We searched the PubMed, the Cochrane Library, Embase, Wanfang database, China National Knowledge Infrastructure (CNKI) database, Chinese Science and Technique Journals (CQVIP), and Chinese Biomedical Database (CBM) for RTCs from the date of their inception to October 10, 2019. Also, we checked whether there was any missing literature accordingly by reading the reference lists of the included systematic reviews. Languages of the trials were restricted to English or Chinese. According to the characteristics of each database, the following keywords were used during the search: (“acupuncture” OR “moxibustion” OR “electroacupuncture” OR “acupoint application” OR “acupoint catgut embedding” OR “Warm Acupuncture”) AND (“functional dyspepsia” OR “dyspepsia”) AND (“Randomized Controlled Trial” OR “Randomized” OR “Randomly”).  Table 1 displays a PubMed search strategy as an example.

### 2.2. Inclusion Criteria

#### 2.2.1. Participants

Patients who were diagnosed with functional dyspepsia (FD), aged 18 years and above and met the criteria of Rome I, II, III, IV or Chinese diagnostic criteria of functional dyspepsia were enrolled regardless of gender or course of the disease. Only patients without any other complications were included.

#### 2.2.2. Interventions and Comparison

In this network meta-analysis, “different acupuncture therapies” were defined as single use of manual acupuncture, appoint catgut embedding, acupoint application, warm acupuncture, moxibustion, and electroacupuncture [30–32] (Table 2).

Our comparison types include different acupuncture treatments compared with each other alone, acupuncture treatments alone compared with western medicine or acupuncture treatments alone compared with sham acupuncture. That is, single use of acupuncture, warm acupuncture, electroacupuncture, moxibustion, acupoint catgut embedding, or acupoint application was considered eligible for this network meta-analysis. The control group was treated with either recommended conventional medicine [9] (proton pump inhibitors (PPIs), histamine-2 receptor antagonists (H2RAs), anticoke depression drugs, protective agents for gastric mucosa, and drugs targeting *H. Pylori*) or sham acupuncture or one of the abovementioned acupuncture options.

Prokinetics available in the market were compared except cisapride. We excluded cisapride as it has been removed from the market due to serious adverse events [33]. Trials evaluating the efficacy of proton pump inhibitors (PPIs) and prokinetics combinations were excluded because a meta-analysis revealed that these combinations have substantial side effects [34].

#### 2.2.3. Outcomes

The primary outcome was an improvement of global symptoms of functional dyspepsia. The outcome of the global assessment on Likert scales has been used for global evaluation [35].

Symptom improvement was evaluated based on short 4-point Likert scales. According to the Cochrane Handbook recommendations [36], the short ordinal outcome results were dichotomized into “improvement” or “no improvement” based on clinical effectiveness. Therefore, in this study, “symptom-free,” “markedly effective,” “effective” were classified as “improvement,” while “Ineffective” was classified as “no improvement.” Likewise, a binary assessment of global symptoms improvement has also been reported [22].

Secondary outcomes were all quality of life measures which included the MOS 36 Item Short-Form Health Survey (SF-36) [37] or Nepean Dyspepsia Life Quality Index (NDLQI) [38]. The number of dropouts, adverse events during, and follow-ups was recorded.

Choices of these outcomes were based on current expert recommendations on endpoints for FD clinical trials [35].

### *2.3*. Exclusion Criteria

1. Duplicate studies
2. Studies without outcome indicators
3. Studies just mentioning “randomly” or with inappropriate random method (order of visits, birthday, and so on)
4. Breastfeeding during pregnancy or a pregnant woman

### 2.4. Study Selection and Data Extraction

All identified studies were imported into NoteExpress. Those that were repeated and did not meet the inclusion criteria were excluded after reading the title and abstract. The final inclusion or exclusion of studies was performed after full-text reading of the eligible studies. Data were extracted by two authors (X. X. Huang, Y. F. Liu) independently. The data extracted included the authors' names, publication date, diagnostic criteria, the number of subjects in the experimental group and the control group, gender and age, course of diseases, intervention and control group type, outcome indicators, and evaluation standard. Any inconsistency in the data extracted was resolved by a third author (Y. L. Hu). Study characteristics (author and year of publication); participant characteristics (diagnose criteria, age, gender, disease course, and cases of each group); intervention information (measures of intervention and control, treatment duration, and adverse events), and outcome (outcomes, numbers of dropouts and adverse events, and follow-ups) were recorded.

### 2.5. Risk of Bias in Individual Studies

Two reviewers (Y. R. Chen, K Lan) assessed the risk of bias of the included RCTs using the Cochrane Collaboration tool for assessing the risk of bias [20]. Each study was evaluated as low, unclean, and high-risk bias based on seven items: (1) random sequence generation; (2) allocation concealment; (3) blinding of participants and personnel; (4) blinding of outcome assessment; (5) incomplete outcome data; (6) selective reporting; and (7) other bias. Any disagreements were resolved by a third reviewer (L. Y. Hu). The risk of bias assessment was summarized using Review Manager 5.3.

### 2.6. Statistical Synthesis

All analyses were performed in Stata/SE (StataCorp/LP, TX, USA) using “mvmeta” and “network” packages in Stata 14.0. Randomized effects model was used for a pairwise meta-analysis for each pair of interventions, using odds ratios (OR) and 95% confidence intervals to estimate relative treatment effectiveness (CI). A network meta-analysis was performed in Stata using the network package [39]. The network evidence graph was implemented through the command “netplot.” Differences between direct and indirect evidence for the same comparisons were first explored, according to the results of *I*^2^ and *p* value of the global; then, the node-splitting analysis method was used to assess the inconsistency between direct and indirect comparisons of each pair of interventions [40]. Finally, the inconsistency factor within each closed loop of evidence was used to detect the inconsistency of direct and indirect comparisons in the same study [41].

We used a comparison-adjusted funnel plot to determine whether there was a small sample effect in the network.

The ranking of the intervention measure results was implemented in Stata 14.0. The surface under the cumulative ranking curves (SUCRA) and mean rank (MR) were used as the evaluation indexes. The SUCRA value was used to reflect the possibility of the intervention measures. The closer MR was to 0, the better the intervention effect [42].

## 3. Results

### 3.1. Characteristics and Risk of Bias of the Included Studies

**Figure 1:**
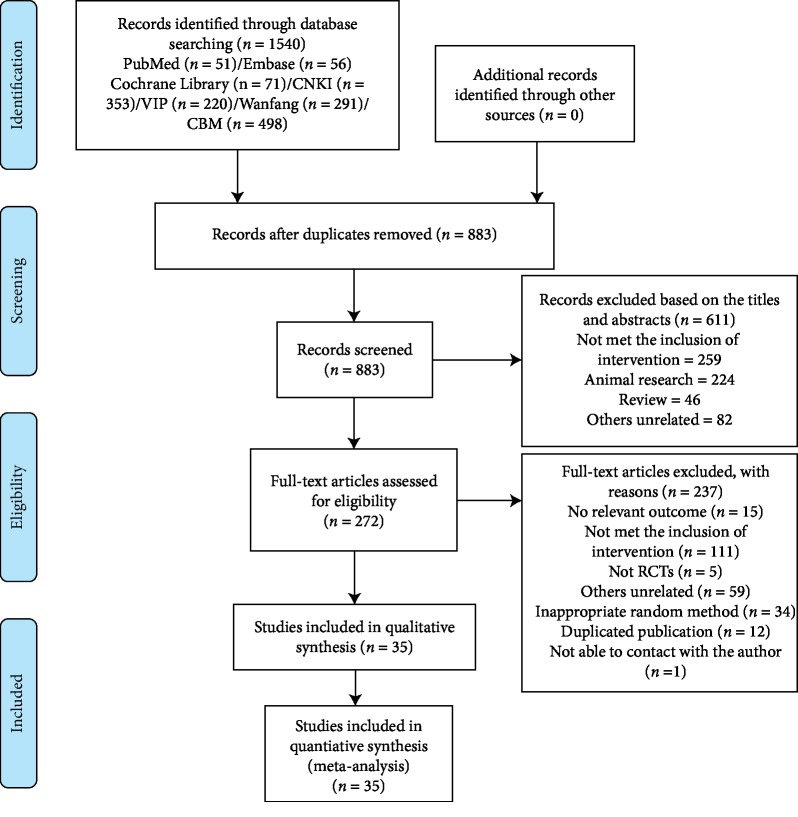
Literature search and selection.

A total of 1540 records were identified. Initially, 657 duplicate records were removed, and 610 records that did not meet the inclusion criteria were removed after reading the article title and abstract. Finally, 272 potentially eligible studies were downloaded based on inclusion and exclusion criteria. Eventually, 35 studies were included in the network meta-analysis (Figure 1).

### 3.2. Study Characteristics

A total of 35 studies (3301 participants) were included in this study, and all were from China, two of the Chinese RCTs are cited by the Science Citation Index (SCI). Thirty-five RCTs were conducted between 2008 and 2018 years. Among the 35 included RCTs, 32 were two-arm trials, whereas three were three arms. Ten interventions were applied, including seven types of acupuncture therapies (acupuncture, electroacupuncture; acupoint application, moxibustion, warm acupuncture; acupoint catgut embedding; sham acupuncture), and three types of oral prokinetics (domperidone, itopride, mosapride). In terms of therapeutic measures, 18 were treated with acupuncture, 9 with electroacupuncture, one with acupoint application, 6 with moxibustion, 2 with acupoint catgut embedding, 2 with warm acupuncture, 7 with sham acupuncture, 13 with domperidone, 10 with itopride, and 4 with mosapride. Concerning diagnostic criteria, 32 RCTs applied the Rome III, one Rome II, 1 Not specified, and one RCT the Chinese criteria.

For outcome indicators, 32 trials included functional dyspepsia outcomes, 12 trials used SF-36 system, and 8 trials used the NDLQI system. The characteristics of included studies are presented in Table 3.

### 3.3. Dropouts, Adverse Event, and Follow-Up

Six trials (22.9%) reported the loss of patients during follow-up and two trials reported adverse reactions. Zou [47] reported that one patient in the moxibustion group was required to withdraw because of a scar and one patient in the acupuncture group was interrupted due to a business trip. Yu [48] reported loss of 14 cases. Adverse reactions occurred in six subjects, representing an incidence rate of 1.69%. The adverse reactions in five of the subjects were related to acupuncture, whereas one subject was not. Zheng [51] reported that four cases of the acupuncture group had blood swelling at acupuncture points. In the conventional western medicine group, one case had a transient rash and two cases had constipation. Yang [57] reported that two cases were lost in the acupuncture group and four cases were lost in the conventional medicine group. In the trial by Zhou et al. [53], four cases were lost; one case in the acupuncture group was not treated because of fear of needles. In the moxibustion group, one case dropped out due to a brain tumor diagnosed during the trial. Hu et al. [63] reported that two cases dropped out in the acupuncture group without a clear reason. Zeng [73] reported 13 cases were lost and five cases were removed (detailed description of the reasons was given), the total loss rate was 3.64%, and the elimination rate was 1.4%. Jin et al. [74] reported four patients (two from each group) dropped out (dropout rate: 6.67%), one patient could not tolerate the acupuncture sensations, and the other patient had transportation difficulties in acupuncture group; the two patients in sham acupuncture group dropped out due to noneffectiveness; Ma et al. [77] reported three cases of acupuncture group dropped out (one patient withdrew due to needle sickness, and two cases could not be treated as prescribed); six cases of sham acupuncture group dropped out (two cases could not be treated as prescribed and three cases did not follow the treatment plan, one patient for personal reasons).

Nine RCTs (25.7%) reported the follow-up, which ranged from one to six months. Jin et al. [49] reported that acupuncture was more effective than conventional medicine in terms of SF36 after two months of follow-up. Fu et al. [54] concluded that the acupuncture group was more effective than the conventional medicine groups after one month of follow-up in terms of patient-reported outcomes (PRO) scale and symptom scores. Cheng [56] reported that the electroacupuncture group had better symptom scores and plasma ghrelin levels than conventional medicine group after three months of follow-ups. The trial by Liu et al. [62] found that acupoint catgut embedding was more effective than conventional western medicine in SF36 and symptom score after 6 months follow-up. Wang et al. [69] found that acupuncture was superior to sham acupuncture in terms of effectiveness rate, symptom score, and quality of life score after 12 weeks and 24 weeks of follow-up. Electroacupuncture was more effective than sham acupuncture in the NDI score after follow-up for 4 weeks and 12 weeks in the trial by Zeng [73]. The SF36 and symptom score after one month and three months follow-up was better in acupuncture and sham acupuncture compared to before treatment [75]. Li [76] showed that the acupuncture improved NDSI and NDLQI at 1 month, 2 months, and 3 months, after 4 and 5 months of follow-up while domperidone treatment did not. Ma et al. [77] showed that the acupuncture more effectively improved NDLQI after treatment and 1 month of follow-up compared with the sham acupuncture group.

Overall, adverse reactions caused by acupuncture were generally not serious and resolved without treatment. Moreover, acupuncture has a long-term effect compared to conventional medicine in improving NDSI, NDLQI, and SF-36 for functional dyspepsia.

### 3.4. Risk of Bias of the Included Studies

**Figure 2:**
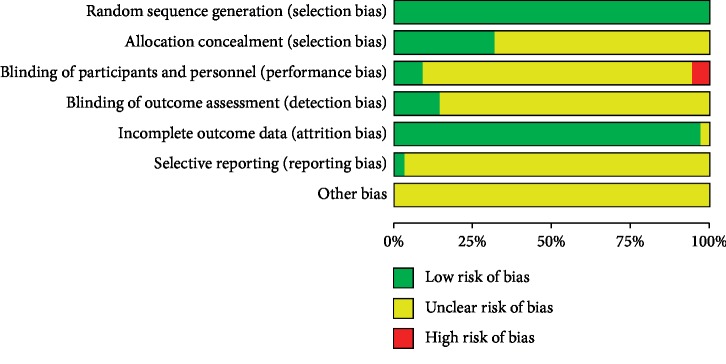
Summary of risk of bias graph.

**Figure 3:**
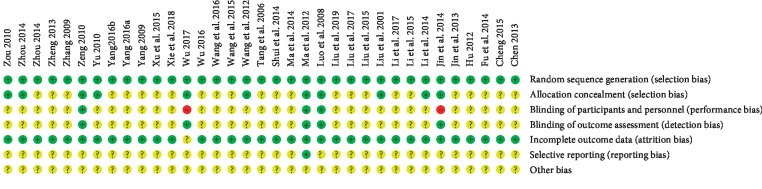
Risk of bias of the included randomized controlled trials.

All the 35 RCTs had a low risk of bias in random sequence generation. In terms of allocation concealment, five trials adopted opaque envelope, five trials used the central randomization, both of which had low risk. In terms of blind methods, four trials adopted blinding of investigators, patients, and outcome evaluators and these trials had low risk. One trial applied the evaluator blinding method and the blinding of investigators and patients was scored as high risk. All included RCTs had a low risk of bias for incomplete data, with eight trials reporting the details of dropouts, which did not affect the outcome. Selective outcome reporting was unclear in all included RCTs except one which provided registration numbers. The risk of bias assessment is summarized in Figures 2 and 3.

### 3.5. Pairwise Meta-Analysis

#### 3.5.1. Effective Rate

See [Supplementary-material supplementary-material-1] in the Supplementary Material for pairwise meta-analysis of an effective rate. Thirteen pairwise meta-analyses were performed to compare the effectiveness of different acupuncture therapies and prokinetics. Detailed results are shown in Table 4.

(i) When compared to domperidone, itopride, mosapride, and sham acupuncture, respectively, manual acupuncture was more effective in alleviating global FD symptoms (6 RCTs, RR: 2.26, 95% CI: 1.76, 6.04, *p*=0.0002, *I*^2^ = 0%) (5RCTs, RR: 7.33, 95% CI: 3.59, 14.95, *p* < 0.00001, *I*^2^ = 0%) (3RCTs, RR: 4.13, 95% CI: 1.81, 9.39, *p*=0.0007, *I*^2^ = 0%) (1RCT, RR: 4.15, 95% CI: 1.37, 12.85, *p*=0.01); (ii) Compared to itopride and sham acupuncture alone, electroacupuncture significantly improved the effectiveness of global FD symptoms (5RCTs, RR: 2.44, 95% CI: 1.63, 3.67, *p* < 0.0001, *I*^2^ = 0%) (4RCTs, RR: 7.55, 95% CI: 5.25, 10.87 *p* < 0.00001, *I*^2^ = 60%); (iii) when compared to domperidone, moxibustion was more effective in alleviating global FD symptoms (3RCTs, RR: 4.13, 95% CI: 2.23, 7.64, *p* < 0.0001, *I*^2^ = 23%); (iv) sham acupuncture was less effective in alleviating global FD symptoms compared to itopride (2RCTs, RR: 0.38, 95% CI: 0.26, 0.56, *p* < 0.00001, *I*^2^ = 0%); there was no statistically significant difference between acupuncture and acupoint application, between acupuncture and moxibustion, between acupoint catgut embedding and domperidone, and between acupoint catgut embedding and itopride in alleviating global FD symptoms.

#### 3.5.2. SF-36

See [Supplementary-material supplementary-material-1] in the Supplementary Material for pairwise meta-analysis of SF-36. Eight pairwise meta-analyses were performed to compare the SF-36 of different acupuncture therapies and prokinetics. Detailed results were shown in Table 5.

(i) When compared to itopride, mosapride, and sham acupuncture, respectively, acupuncture was more effective in improving SF-36 (4RCTs, SMD: 8.82, 95% CI: 5.62, 12.02 *p* < 0.00001, *I*^2^ = 46%), (1RCT, SMD:6.20, 95% CI: 0.42, 11.98 *p*=0.04), (1RCT, SMD: 14, 95% CI: 8.20, 19.80 *p* < 0.00001); (ii) electroacupuncture was more effective in improving SF-36 compared to itopride (1 RCT, SMD: 14.35, 95% CI: 3.79, 24.91 *p*=0.008) and sham acupuncture (2RCTs, SMD: 17.84, 95% CI: 15.31, 20.36 *p* < 0.00001); (iii) acupoint catgut embedding was more effective in improving SF-36 compared to domperidone (1RCT, SMD:9.90, 95% CI: 1.01, 18.79 *p*=0.03); the remaining two pairs (between acupuncture and domperidone, between acupoint catgut embedding and mosapride) were not statistically different.

#### 3.5.3. NDLQI

See [Supplementary-material supplementary-material-1] in the Supplementary Material for pairwise meta-analysis of NDLQI. Eight pairwise meta-analyses were performed to compare the NDLQI of different acupuncture therapies and prokinetics. Detailed results were shown in Table 6.

(i) When compared to domperidone, itopride, and sham acupuncture alone, acupuncture was more effective in improving NDLQI (1RCT, SMD:11.09, 95% CI: 8.17, 13.47 *p* < 0.00001) (1RCT, SMD: 10.82, 95% CI: 5.61, 16.03 *p* < 0.0001) (1RCT, SMD: 17.00, 95% CI: 12.53, 21.47 *p* < 0.00001); (ii) when compared to domperidone, itopride, and sham acupuncture alone, electroacupuncture was more effective in improving NDLQI (1RCTs, SMD: 3.2, 95% CI: 0.60, 5.8 *p*=0.02) (1RCT, SMD: 3.02, 95% CI: 0.07, 5.97 *p* = 0.04) (3RCTs, SMD: 7.31, 95% CI: 5.65, 8.96 *p* < 0.0001, *I*^2^ = 0%); (iii) itopride was more effective in improving NDLQI compared to sham acupuncture (1RCT, SMD: −4.46, 95% CI: −7.35, −1.57, *p*=0.003); the remaining one pair (between acupuncture and moxibustion) was not statistically different.

### 3.6. Network Meta-Analysis

#### 3.6.1. Network Map for Different Interventions

**Figure 4:**
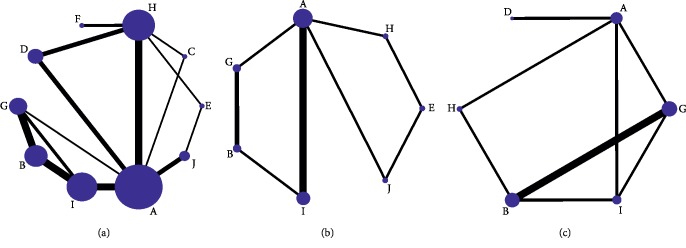
Network meta-analysis of eligible comparisons. (a) Effective rate. (b) SF-36. (c) NDLQI. Note: A: acupuncture; B: electroacupuncture; C: acupoint application; D: moxibustion; E: acupoint catgut embedding; F: warm acupuncture; G: sham acupuncture; H: domperidone; I: itopride; J: mosapride.

Thirty-three studies covering ten interventions and 3098 patients were merged for analysis of effective rate (Figure 4(a)), SF-36 was reported in 12 studies including 759 patients and seven interventions (Figure 4(b)), and NDLQI was reported in 8 studies involving 1007 patients and six interventions (Figure 4(c)). The thickness of the lines in the figure represents the number between the two interventions. The size of the nodes in the figure represents the sample number (Figure 4).

#### 3.6.2. Evaluation of Statistical Inconsistency

The inconsistency of test results of effective rate, SF-36, and NDLQI (*p*=0.3641, 0.9043, 0.9582 > 0.05) was not in statistically significant heterogeneity with the data, so the consistency model was selected. All local inconsistency tests were performed with node-splitting method. See Tables [Supplementary-material supplementary-material-1]–[Supplementary-material supplementary-material-1] in the Supplementary Material for inconsistency tests based on side-splitting approach. Inconsistency tests for all nodes showed *p* values were greater than 0.05, indicating that there was no significant difference between direct and indirect comparison. See Figures [Supplementary-material supplementary-material-1]–[Supplementary-material supplementary-material-1] in the Supplementary Material for inconsistency tests based on loop-specific approach. All the results of the closed-loop inconsistency showed that 95% CI contained 0, and all IF were close to zero, indicating that there was no apparent inconsistency in the comparisons of closed circles.

#### 3.6.3. Comparative Results of Different Interventions

*(1) Effective Rate*. A league table was established to compare efficacy among ten interventions (32 trials) for patient-reported global FD symptom (Table 7).

(i) Compared with sham acupuncture, nine interventions were significantly more effective (last row of Table 7); (ii) compared with mosapride, eight interventions were significantly more effective except domperidone and sham acupuncture (second last row of Table 7); (iii) compared with domperidone, five acupuncture therapies were significantly more effective except electroacupuncture (third last row of Table 7); (iv) acupuncture was more effective than mosapride (first column of Table 7); the rest were not significantly different.

See [Supplementary-material supplementary-material-1] and [Supplementary-material supplementary-material-1] in the Supplementary Material for ranking probabilities for effectiveness rate. Acupuncture had the best efficacy (81.6%), followed by acupoint application (74.0%), warm acupuncture (71.6%), moxibustion (71.5%), acupoint catgut embedding (70.4%), electroacupuncture (53.2%), mosapride (42.4%), domperidone (21.9%), itopride (13.3%), and sham acupuncture (0%).

*(2) SF-36*. Twelve trails, including seven interventions, are reported on SF-36 (Table 8). The results showed that (i) electroacupuncture effectively improved the life quality of FD patients compared to sham acupuncture, domperidone, and itopride alone (first column of Table 8); (ii) acupuncture was more effective in improving SF-36 compared to itopride and sham acupuncture alone (second column of Table 8).

See [Supplementary-material supplementary-material-1] and [Supplementary-material supplementary-material-1] in the Supplementary Material for ranking probabilities of SF-36. Electroacupuncture (91.8%) had the best effect in improving SF-36, followed by acupuncture (77.3%), acupoint catgut embedding (71.7%), mosapride (46.0%), itopride (30.2%), domperidone (23.2%), and sham acupuncture (9.8%).

*(3) NDLQI*. Eight trails, including six interventions reported on NDLQI (Table 9). (i) The results showed that electroacupuncture, moxibustion, acupuncture, electroacupuncture, domperidone, and itopride were more effective in improving NDLQI compared to sham acupuncture (last row of Table 9); (ii) electroacupuncture and moxibustion more effectively improved NDLQI compared to itopride and domperidone alone (second and third last row of Table 9); (iii) acupuncture more effectively improved NDLQI compared to electroacupuncture (second row of Table 9).

See [Supplementary-material supplementary-material-1] and [Supplementary-material supplementary-material-1] in the Supplementary Material for ranking probabilities of NDLQI. Moxibustion (93.2%) had the best effect in improving NDLQI, followed by acupuncture (85.4%), electroacupuncture (57.3%), domperidone (33.2%), itopride (30.0%), and sham acupuncture (0.9%).

#### 3.6.4. Small Sample Size Effect

See Figures [Supplementary-material supplementary-material-1]-[Supplementary-material supplementary-material-1] in the Supplementary Material for comparison-adjusted funnel plots. There was no strong evidence of small study effects across outcomes except for one study of moxibustion which had a large efficiency. Because there are fewer than ten studies using NDLQI as the outcome indicator, no funnel plot was performed.

## 4. Discussion

In this review, 35 studies covering 3301 patients diagnosed with functional dyspepsia were enrolled. The results of network meta-analysis show that manual acupuncture, electroacupuncture, acupoint application, moxibustion, acupoint catgut embedding alone, and warm acupuncture alone are more effective in improving the global symptoms of functional dyspepsia compared with three kinds of prokinetics (domperidone, itopride, and mosapride) and sham acupuncture. Moreover, manual acupuncture produced the best results. In terms of improving SF-36, both electric acupuncture alone and manual acupuncture alone were more effective than itopride, and electroacupuncture alone was superior to domperidone. In terms of improving NDLQI, acupuncture alone and moxibustion alone produced superior effects than itopride alone and domperidone alone. Furthermore, acupuncture therapy was more effective than conventional medicine in improving the overall symptoms and quality of life of patients, even during the follow-up period of 1 to 6 months.

This review enrolled 31 RCTs diagnosed with Rome III, and one diagnosed with Rome II. The Rome III criteria have been used to diagnose FD since it was introduced in 2006. It only differs from Rome II in the course of the disease. In Rome III criteria, the total course of the disease was shortened from one year to six months. In addition, FD is classified into postprandial distress syndrome (PDS) and epigastric pain syndrome (EPS) based on meals in Rome III criteria. It has, therefore, been reported that Rome III yields higher rates of diagnosis than Rome II [79].

In May 2016, a new Rome IV criterion was established based on the increasing understanding of FD in the past decade [1]. In the Rome IV criteria, minor modifications were made to consider postprandially occurring symptoms as part of PDS [1, 80]. Therefore, our findings may also apply to patients diagnosed with Rome IV criteria. A recent study by Aziz and colleagues suggested that the subtypes of functional dyspepsia based on criteria for Rome IV have different pathophysiological mechanisms or influences [81]. Thus, future studies should investigate the effects and mechanisms of different acupuncture therapies in line with the Rome IV functional dyspepsia subtypes.

The results of this systematic review are in agreement with previous reviews [19–22, 82] except for one [83]. In a Cochrane review [83] of four trials, acupuncture was not significantly different from other medications in reducing FD symptoms. Of note, in our analysis, we excluded the trial by Shi et al. 2009 [84] as they used cisapride in the control group and Zhou and Zheng 2005 [85] as they did not provide clear randomization methods. This may explain the discrepancy in the results. Although other systematic reviews have reported that acupuncture therapy is more effective than conventional medicine, they all mentioned that the methodologies of the included studies are flawed. Therefore, further high-quality acupuncture studies are required to provide more convincing results.

In recent years, advances in acupuncture research have led to a better understanding of the mechanisms of acupuncture on functional dyspepsia. Such mechanisms are divided into five aspects: (1) Electrophysiology mechanism: One study showed that transcutaneous electroacupuncture at the acupoints of ST36 and PC6 increased the amplitude and regularity of slow waves in patients with FD, which promotes gastrointestinal motility [86]. It has also been demonstrated that [26] moxibustion on Zusanli(ST36) regulates abnormal gastric electrogastrogram in FD patients. In this way, it improves the symptoms of FD, with an effective rate of up to 97%. (2) Imaging mechanism: One RCT showed that compared to healthy subjects, acupuncture stimulation at ST36 with fMRI technique evoked pronounced changes in the homeostatic afferent processing network of FD patients [87]. Fang et al. [88] found that acupuncture relieved gastrointestinal symptoms and signs using resting-state fMRI, which may be due to the normalization of brain-gut axis in FD patients. (3) Molecular mechanism: A study performed by Zhang and Guo showed that EA increased plasma motilin, thereby promoting gastric emptying and alleviating the symptom of dyspepsia [89]. (4) Metabolomics mechanisms: Li et al. [90] found that stimulation of different acupoints regulates the plasma levels of markers of FD as revealed by high performance liquid chromatography-mass spectrometry combined with metabolomics. (5) Genomics mechanism: Research has found that electroacupuncture stimulation at ST36 regulates gastric motility by activating PKC and MAPK signal transduction pathways [91]. Electroacupuncture inhibits MEK/ERK1/2 signaling pathway and restores the enteric nervous system in FD rats [92].

This study has the following limitations. First, all subjects in the 35 RCTs were Chinese, and thus the results obtained in this study are not to be applicable to patients in other countries. In addition, some studies did not provide details concerning the application of acupuncture, which limits strength of our conclusions. We attempted to describe in detail the acupoints and methods used as much as possible. We recommend that future clinical trials on acupuncture should follow the STRICTA standard [93] and involve subjects from other countries and regions. Second, although we excluded studies in which randomization methods were inappropriate or unclear, the risk of bias for allocation concealment, blindness, and selective outcome reporting is unclear in most of the included studies. This may reduce the credibility of our conclusions. Given that the reported outcomes of this study are subjective symptom scores, blinding of outcomes evaluators should be applied in RCTs to reduce bias and increase the reliability of the results. In the future, researchers should follow the CONSORT reporting statement [94] to improve reporting quality. Third, of the 35 included studies, 31 used dichotomous of symptom relief as endpoint, and only 12 and 8 studies reported SF-36 and NDIQI, respectively. Although the dichotomous endpoint of reported symptoms alleviation is commonly used as the primary endpoint of functional dyspepsia, it has low sensitivity and validity [38]. More patient-reported outcomes should be added in future trials, such as disease-specific questionnaires and quality of life assessments, to comprehensively evaluate the treatment effects.

## 5. Conclusion

These direct comparisons and network meta-analysis revealed that manual acupuncture alone can effectively improve FD symptoms, hence may be used for FD patients who do not respond to prokinetics or cannot tolerate adverse effects of prokinetics. However, further multiple centers and high-quality RCT studies should be conducted to confirm the findings of this study.

## Figures and Tables

**Table 1:** The search strategy for PubMed.

Search	Query	Item found	Time
#31	Search #7 AND #20 AND #29	51	7:02:01
#30	Search #27 AND #28	1963308	7:01:02
#29	Search “humans” [mesh]	18204958	6:59:31
#28	Search #21 OR #22 OR #23 OR #24 OR #25 OR #26 OR #27	2912831	6:58:06
#27	Search groups [title/abstract	1994835	6:57:33
#26	Search trial [title/abstract]	567330	6:57:19
#25	Search randomly [title/abstract]	321036	6:56:59
#24	Search placebo [title/abstract]	194510	6:56:50
#23	Search randomized [title/abstract]	495114	6:56:43
#22	Search controlled clinical trial [publication type]	581079	6:56:12
#21	Search randomized controlled trial [publication type]	492614	6:55:26
#20	Search #8 OR #9 OR #10 OR #11 OR #12 OR #13OR #14 OR #15 OR #16 OR #17 OR #18 OR #19	24791	6:53:56
#19	Search catgut implantation [title/abstract]	43	6:51:56
#18	Search acupoint catgut embedding [title/abstract]	87	6:51:34
#17	Search acupoint sticking therapy [title/abstract]	32	6:50:43
#16	Search acupoint application [text word]	58	6:49:51
#15	Search warm needle acupuncture	61	6:49:12
#14	Search needle warming therapy [title/abstract]	3	6:48:41
#13	Search warm acupuncture [title/abstract]	34	6:46:58
#12	Search moxibustion [title/abstract]	2432	6:46:23
#11	Search electroacupuncture [title/abstract]	4197	6:46:08
#10	Search acupuncture therapy [title/abstract]	1060	6:45:32
#9	Search acupuncture [title/abstract]	21116	6:44:59
#8	Search “Acupuncture” [mesh]	1637	6:44:39
#7	Search #1 OR #2 OR #3 OR #4 OR #5 OR #6	10741	6:43:48
#6	Search nonulcer dyspepsia [title/abstract]	1063	6:43:18
#5	Search functional dyspepsia [title/abstract]	3026	6:40:48
#4	Search indigestions [title/abstract]	6	6:40:21
#3	Search indigestion [title/abstract]	993	6:40:01
#2	Search dyspepsia [title/abstract]	57	6:38:22
#1	Search “dyspepsia” [mesh]	8467	6:37:31

**Table 2:** Definitions of different acupuncture therapies.

Type of acupuncture	Definitions
Manual acupuncture	Needle insertion into acupuncture points, followed by manual manipulation. Needling promotes Qi (the vital energy) in the meridians.
Appoint catgut embedding	An acupuncture technique that involves weekly infixing of surgical chromic catgut sutures into the subcutaneous tissue of acupoints located at the abdomen, extremities, and the back with a specialized needle under aseptic precautions.
Acupoint application	Acupoint application in which Chinese medicine is applied to an acupoint of the human body. This method has both the acupoint stimulation and pharmacological effects.
Warm acupuncture	A method combining acupuncture with moxibustion, a moxa column is added to the end of the needle, ignites it, and the heat is transmitted through the needle to the body after being ignited to treat diseases.
Moxibustion	A method in which a moxa herb is burned above the skin or on the acupuncture points. It can be used as a cone stick, loose herb, or applied at the end of the acupuncture needles. The purpose of moxibustion is to apply heat to the acupuncture points to alleviate symptoms.
Electroacupuncture	A modern acupuncture technique used with manual acupuncture, where a needle is attached to a trace pulse current after it is inserted to the selected acupoint to produce a synthetic effect of electric and needling stimulation.

**Table 3:** Characteristics of included.

Studies (year)	Multicenter clinical study (Yes/No)	Diagnostic criteria	Patient admission time	No. of participants(T/C)(M/F)	Mean age (years) (T/C)	Course of disease (T/C)	Intervention group (main acupuncture points)	Frequency and treatment course	Control group	Outcome
Xu et al. 2015 [43]	No	Rome III	From March 2012 to February 2014	(20/27)/(21/25)	(38.76 ± 9.52)/(39.74 ± 10.28)	(2.42 ± 0.53) y/(2.01 ± 0.19) y	MA (PC6, ST36, ST25 of both sides; CV6, CV10, CV12, CV13, EX-HN3, DU24, DU20)	Once daily, 5/w for 4 w	Domperidone (10 mg taken orally three times a day, 15 min before each meal) 4 w	Categorical, NDLQI
Tang et al. 2006 [44]	No	Rome II	Not specified	(15/17)/(14/16)	(38.2 ± 11.3)/(36.7 ± 12.8)	(3 m–10 y)/(3 m–9 y)	MA (ST36, ST44, LR3, PC6, BL20, BL21, BL18 of both sides; CV12)	Once daily for 3 courses; 10 d/course	Domperidone (10 mg taken orally three times a day, 30 min before each meal) for 30 days	Categorical
Luo 2008 [45]	No	Rome III	From December 2006 to December 2007	(15/15)/(17/13)	(46.31 ± 10.43)/(42.13 ± 12.3)	(26.88 ± 6.18) m/(24.23 ± 6.79) m	M (CV12)	Once daily for 4 w	MA(CV12) for 4 w	Categorical
Yang 2009 [46]	No	Rome III	From April 2008 to January 2009	(13/11)/(14/10)	(35.70 ± 10.43)/(35.23 ± 11.25)	(18.40 ± 12.73)m/(18.17 ± 13.55)m	EA(CV12, BL12, ST36)	Once daily, 5/w for 4 w	Itopride hydrochloride tablets (50 mg taken orally three times a day, 30 min before each meal) for 4 w	Categorical
Zou 2010 [47]	No	Rome III	From January 2009 to January 2010	(13/16)/(14/15)	(38.28 ± 11.53)/(41.38 ± 9.49)	(57.90 ± 19.17) m/(57.45 ± 14.87) m	M(CV12, ST36, BL26, BL21, SP6, BL20)	Once daily, 6/w for 2 w	MA(CV12, ST36, BL26, BL21, SP6, BL20) Once daily, 6/w for 2 w	Categorical
Yu 2010 [48]	Yes	Rome III	From April 2008 to August 2009	(33/82)/(34/83)/(36/81)	(36.68 ± 13.51)/(37.18 ± 13.03)/(37.32 ± 13.79)	(74.50 ± 74.16) m/(64.88 ± 75.81) m/(66.31 ± 66.60) m	EA (ST42, ST40, ST36, ST34 of both sides)	Once daily, 5/w for 4 w	Sham EA (nonacupoints, three in upper limbs and one near ST36) itopride hydrochloride tablets (50 mg taken orally three times a day, 30 min before each meal) for 4 w	Categorical, NDLQI
Jin et al. 2013 [49]	No	Rome III	From March 2011 to March 2012	(12/22)/(16/20)	(45.21 ± 9.37)/(44.81 ± 8.95)	(23.68 ± 14.66) m/(23.89 ± 13.13) m	MA(CV12, ST36, SP6, ST25 of both sides)	Once daily, 6/w for 2 w	Itopride hydrochloride tablets (50 mg taken orally three times a day, 30 min before each meal) for 2 w	Categorical, SF-36
Chen 2013 [50]	No	Rome III	From July 2011 to February 2013	(12/18)/(14/16)	(45.3 ± 11.8)/(46.2 ± 12.3)	(20.5 ± 7.8) m/(20.6 ± 7.6) m	MA (CV12; ST36, PC6, LR3 of both sides; CV17)	Once daily, 5/w for 4 w	Itopride hydrochloride tablets (50 mg taken orally three times a day, 30 min before each meal) for 4 w	Categorical, SF-36
Zheng 2013 [51]	No	Rome III	Not specified	(14/16)/(13/17)	(34.77 ± 10.25)/(34.03 ± 8.97)	(18.50 ± 8.92) m/(21.13 ± 9.98) m	WA (CV12, ST36, SP6)	Once daily, for 4 w	Domperidone (10 mg taken orally three times a day, 30 min before each meal) for 4 w	Categorical
Shui et al. 2014 [52]	No	Rome III	From October 2010 to January 2013	(22/18)/(19/21)	42/41	(6 m–10.5 y)/(7.5 m–12y)	MA (SP6, SP9, SJ6 of both sides)	Once daily, 7/w for 3 w	Mosapride citrate dispersible tablets (5 mg taken orally three times a day) for 3 w	Categorical
Zhou et al. 2014 [53]	No	Rome III	From March 2011 to September 2012	(12/22/(16/20)	(45 ± 9)/(45 ± 9)	(23.68 ± 14.65) m/(23.89 ± 13.13) m	MA(CV12, ST36, ST25, SP6)	Once daily, 6/w for 2 w	Itopride hydrochloride tablets (50 mg taken orally three times a day, 30 min before each meal) for 2 w	Categorical, SF-36
Fu et al. 2014 [54]	No	Rome III	From December 2011 to June 2013	(19/21)/(17/23)/(18/22)	(40.03 ± 9.37)/(41.47 ± 10.56)/(40.89 ± 9.76)	(17.08 ± 12.97) m/(18.42 ± 9.37) m/(17.97 ± 10.28) m	AA(CV12, ST36, ST25, SP6)	Once daily, 6/w for 4 w	Itopride hydrochloride tablets (50 mg taken orally three times a day, 30 min before each meal) for 4 w	Categorical
Liu 2015 [55]	No	Rome III	From May 2013 to May 2014	(19/15)/(18/16)	(38 ± 9)/(38 ± 9)	(15.54 ± 6.77) m/(14.82 ± 7.31) m	MA (CV12; PC6, LR14, ST25, ST36, LR3, LR2 of both sides)	Once daily, 7/w for 2 w	Domperidone (10 mg taken orally three times a day, 30 min before each meal) for 2 w	Categorical
Cheng 2015 [56]	No	Rome III	During the period of 2014	(8/12)/(11/9)	(51.60 ± 12.05)/(52.65 ± 11.33)	(4.83 ± 0.97) m/(4.72 ± 1.19) m	EA(CV12, BL12, ST36, LR3, RN17)	Once daily, 6/w for 2 w	Itopride hydrochloride tablets (50 mg taken orally three times a day, 30 min before each meal) for 2 w	Categorical
Yang 2016 [57]	No	Rome III	From June 2014 to August 2015	(7/11)/(7/9)	(40.26 ± 10.9)/(40.41 ± 9.33)	(21.02 ± 9.67) m/(20.38 ± 10.52) m	MA(ST36, CV12, ST25)	Once daily, 6/w for 4 w	Domperidone (10 mg taken orally three times a day, 30 min before each meal) for 4 w	Categorical, SF-36
Li and Tan 2017 [58]	No	Rome III	From December 2013 to December 2015	(33/19)/(30/21)	(82.2 ± 6.8)/(82.8 ± 7.1)	(1.8 ± 0.5) y/(1.9 ± 0.4) y	MA (BL20, BL21, ST36, RN11, ST23)	Once daily, 7/w for 4 w	Mosapride citrate dispersible tablets (5 mg taken orally three times a day) for 4 w	Categorical, SF-36
Liu et al. 2017 [59]	No	Rome III	Not specified	24/24	(10–50)	(0.5–7) y	MA(SP14, ST42)	Once daily, 7/w for 4 w	Mosapride citrate dispersible tablets (5 mg taken orally three times a day) for 4 w	Categorical
Wu 2017 [60]	No	Rome III	From July 2015 to February 2017	(12/13)/(11/14)	(42.14 ± 11.29)/(41.35 ± 12.01)	(36.34 ± 13.24) m/(34.68 ± 12.80) m	ACE(CV12, ST36, ST25, BL18, BL20)	Once daily for 3 courses; 10 d/course	Domperidone (10 mg taken orally three times a day, 30 min before each meal) for 30 d	Categorical, SF-36
Zhou 2014 [61]	No	Rome III	From April 2015 to April 2016	(10/19)/(4/23)	(30.3 ± 12.16)/(31.48 ± 13.29)	(37.31 ± 39.18) m/(38.2 ± 34.7) m	MA(ST36, CV12)	Once daily, 5/w for 4 w	M(ST36, CV12) for 4 w	LDQ, NDLQI
Liu et al. 2019 [62]	No	Rome III	From September 2016 to March 2018	(11/19)/(18/12)	(42.30 ± 11.57)/(40.83 ± 11.50)	(1.5 ± 0.78) y/(0.9 ± 0.56) y	ACE (CV12, ST36, BL21)	Once daily for 4 courses; 15 d/course	Mosapride citrate dispersible tablets (5 mg taken orally three times a day) for 4 w	Categorical, SF-36
Hu 2012 [63]	No	Rome III	From July 2017 to October 2018	(12/22)/(16/20)	(45.21 ± 9.37)/(44.81 ± 8.95)	(23.68 ± 14.65) m/(23.89 ± 13.13) m	EA (CV12; ST25, PC6, ST36 of both sides)	Once daily, 5/w for 4 w	Itopride hydrochloride tablets (50 mg taken orally three times a day, 30 min before each meal) for 4 w	Categorical, SF-36, NDLQI
Wu 2016 [64]	No	Rome III	From January 2014 to January 2015	(24/33)/(23/34)	(45.62 ± 5.14)/(45.48 ± 5.09)	(21.54 ± 6.54) m/(21.61 ± 6.48) m	MA (CV12, SP6, ST36, LR3)	Once daily, 5/w for 4 w	Itopride hydrochloride tablets (50 mg taken orally three times a day, 30 min before each meal) for 4 w	Categorical
Liu et al. 2001 [65]	No	The clinical classification of dyspepsia, 1995	Not specified	(21/17)/(13/17)	(43.6 ± 14.7)/(42.9 ± 14.6)	3.16/3.21	MA(CV12, ST36, PC6, L14, BL21, BL20, LR3, CV6, CV4, ST25)	Once daily, 30 times for 34 day	Domperidone (10 mg taken orally three times a day) for one month;	Categorical
Yang 2016 [66]	No	Not specified	From April 2015 to April 2016	(26/29/(25/30)	(39.4 ± 2.6)/(39.7 ± 2.1)	Not specifified	WA (CV12, ST36, ST25)	Once daily, 7/w for 4 w	Domperidone (10 mg taken orally three times a day) for one month;	Categorical
Ma et al. 2012 [67]	Yes	Rome III	From April 2008 to October 2009	(23/83)/(34/84)/(37/82)	(38.1 ± 13.5/(36.8 ± 13.1)/(36.2 ± 13.9)	(74.2 ± 73.9) m/(64.4 ± 75.6) m	EA (CV12; ST25, ST36 of both sides)	Once daily, 6/w for 2w	Sham EA (nonacupoints, near the main acupoints of EA group) for 2 w	SID, 36
Zhang 2009 [68]	No	Rome III	From April 2008 to January 2009	(13/11)/(14/10)	(35.70 ± 10.43)/(35.23 ± 11.25)	(18.40 ± 12.73) m/(18.17 ± 13.55) m	EA (CV12, BL12, ST36)	Once daily, 5/w for 4 w	Itopride hydrochloride tablets (50 mg taken orally three times a day, 30 min before each meal) for 4 w	Categorical, SF-36
Wang et al. 2015 [69]	No	Rome III	From December 2012 to December 2013	(10/24)/(8/26)	41.8/44.6	5.48 y/8.29 y	MA (ST36, PC5)	Once daily, 5/w for 4 w	Sham MA (nonacupoints, near the main acupoints of EA group) for 4 w	Categorical, NDLQI
Wang et al. 2016 [70]	No	Rome III	From April 2014 to March 2015	(13/15)/(16/12)	(38 ± 15)/(38 ± 14)	(22.35 ± 10.83) m/(22.39 ± 11.91) m	MA (ST20, ST21, SP4, BL20, CV6)	Once daily, 6/w for 2 w	Domperidone (10 mg taken orally three times a day, 30 min before each meal) for 2 w	NDI
Li et al. 2015 [71]	No	Rome III	From May 2013 to May 2014	(37/24)/(36/25)	(64 ± 5.83)/(66 ± 6.49)	Not specifified	M (CV12, CV8, ST25, ST36, BL20, BL18, BL17, SP6 of both sides)	Once daily, 7/w for 4 w	Domperidone (10 mg taken orally three times a day, 30 min before each meal) for 4 w	Categorical
Xie and Xu 2018 [72]	No	Rome III	From July 2016 to July 2017	(56/44)	(44 ± 11)/(43 ± 11)	(7.5 ± 1.5) y/(7.6 ± 1.9)	M (CV8, CV12)	Once daily, 7/w for 4 w	Domperidone (10 mg taken orally three times a day, 30 min before each meal) for 4 w	Categorical
Zeng 2010 [73]	Yes	Rome III	From April 2008 to October 2009	(33/83)/(34/84)	(38.11 ± 13.46)/(36.76 ± 13.10)	(76.47 ± 75.02) m/(65.43 ± 76.36) m	EA(ST34, ST42, ST40)	Once daily, 5/w for 4 w	Sham MA (nonacupoints, near the main acupoints of EA group) for 4 w	Categorical, NDLQI
Jin et al. 2014 [74]	No	Rome III	From July, 2010 to January 2011	(11/17)/(10/18)	(49.32 ± 10.29)/(48.25 ± 11.40)	(12.20 ± 12.20) y/(12.11 ± 10.20) y	MA (ST36, KI3, GB4, PC6, HT7)	Every other day, 3-4/w for 4 w	Sham MA (nonacupoints, different area of innervation with the main acupoints of MA group) for 4 w	SF-36
Wang et al. 2012 [75]	Yes	Rome III	From April 2008 to December 2009	(14/22)/(12/29)	(33.05 ± 1.22)/(32.56 ± 1.20)	(66.58 ± 5.16) m/(47.29 ± 4.04) m	EA(ST42, ST36, ST40, ST34)	Once daily, 5/w for 4 w	Sham EA (nonacupoints, near the main acupoints of MA group) for 4 w	SF-36
Li 2014 [76]	No	Rome III	From September 2012 to July 2013	(12/23/(13/23)	(33.05 ± 1.22)/(32.56 ± 1.21)	(6.35 ± 8.09) y/(8.55 ± 9.59) y	EA (ST36, PC6 of one side; switch the sides each time)	Once daily, 5/w for 4 w	Sham EA (nonacupoints, three points in upper limbs and one near ST36) for 4 w	NDLQI
Ma 20 14 [77]	No	Rome III	From January 2011 to December 2012	(17/15)/(13/16)	(36 ± 5)/(34 ± 5)	(45 ± 7.1) m/(46 ± 8.8) m	EA (CV12; ST25, ST36 of both sides)	Once daily, 6/w for 2 w	Sham EA (nonacupoints, near the main acupoints of EA group) for 2 w	Categorical, SF-36, NDLQI

Categorical: For 28 of these trials [43–60, 62, 63, 65, 66, 68, 69, 71–73, 77], the effectiveness rate was calculated by China's clinical effective standards; NDI, SID, LDQ in the table represents a measure of efficiency using this index. Effectiveness rate = (total points of symptom before treatment − total points of symptom after treatment)/total points of symptom before treatment × 100%. The total effective rate was calculated for the number of cured and improved cases; EA: electroacupuncture; MA: manual acupuncture; WA: warm acupuncture; AA: acupoint application; ACE: acupoint catgut embedding; M: moxibustion; NDI: nepean dyspepsia index; SID: symptom index of dyspepsia; SF-36: the MOS item short from health survey, SF-36; NDLQI: Nepean Dyspepsia Life Quality Index; m: month; y: year; w: week; d: day; M: male, F: Female.

**Table 4:** Pairwise meta-analysis of effective rate.

Comparison	Number	OR (95% CI)	*p*	*I* ^2^ (%)
A VS H	6	3.26 (1.76, 6.04)	0.0002	0
A VS I	5	7.33 (3.59, 14.95)	<0.00001	0
A VS J	3	4.13 (1.81, 9.39)	0.0007	0
A VS G	1	4.15 (1.37, 12.58)	0.01	—
A VS C	1	1.00 (0.23, 4.31)	1	—
A VS D	3	1.94 (0.62, 6.02)	0.25	0
B VS I	5	2.44 (1.63, 3.67)	<0.0001	0
B VS G	4	7.55 (5.25, 10.87)	<0.00001	60
D VS H	3	4.13 (2.23, 7.64)	<0.00001	23
G VS I	2	0.38 (0.26, 0.56)	<0.00001	0
E VS H	1	7.58 (0.84, 68.46)	0.07	—
E VS J	1	1.38 (0.28, 6.80)	0.69	—

*Note.* A: acupuncture; B: electroacupuncture; C: acupoint application; D: moxibustion; E: acupoint catgut embedding; F: Warm acupuncture; G: sham acupuncture; H: domperidone; I: itopride; J: mosapride.

**Table 5:** Pairwise meta-analysis of SF-36.

Comparison	Number	SMD (95% CI)	*p*	*I* ^2^ (%)
A VS I	4	8.82 (5.62, 12.02)	<0.00001	46
A VS J	1	6.20 (0.42, 11.98)	0.04	—
A VS H	1	8.75 (−0.66, 18.16)	0.07	0
A VS G	1	14.00 (8.20, 19.80)	<0.00001	—
B VS I	1	14.35 (3.79, 24.91)	0.008	—
B VS G	2	17.84 (15.31, 20.36)	<0.00001	91
E VS J	1	3.60 (−3.59, 10.79)	0.33	—
E VS H	1	9.90 (1.01, 18.79)	0.03	—

*Note.* A: acupuncture; B: electroacupuncture; E: acupoint catgut embedding; G: sham acupuncture; H: domperidone; I: itopride; J: mosapride.

**Table 6:** Pairwise meta-analysis of NDLQI.

Comparison	Number	SMD (95% CI)	*p*	*I* ^2^ (%)
A VS H	1	11.09 (8.71, 13.47)	<0.00001	—
A VS I	1	10.82 (5.61, 16.03)	<0.0001	—
A VS G	1	17.00 (12.53, 21.47)	<0.00001	—
A VS D	1	−2.68 (−9.18, 3.82)	0.42	—
B VS G	3	7.31 (5.65, 8.96)	<0.00001	0
B VS H	1	3.20 (0.60, 5.80)	0.02	—
B VS I	1	3.02 (0.07, 5.97)	0.04	—
G VS I	1	−4.46 (−7.35, −1.57)	0.003	—

*Note.* A: acupuncture; B: electroacupuncture; D: moxibustion; G: sham acupuncture; H: domperidone; I: itopride.

**Table 7:** League Table of effective rate.

A									
1.01 (0.88, 1.16)	C								
1.01 (0.87, 1.18)	1.00 (0.82, 1.22)	F							
1.02 (0.95, 1.09)	1.01 (0.87, 1.17)	1.01 (0.86, 1.17)	D						
1.02 (0.87, 1.19)	1.01 (0.82, 1.24)	1.01 (0.82, 1.24)	1.00 (0.85, 1.18)	E					
1.07 (0.96, 1.20)	1.06 (0.89, 1.27)	1.06 (0.88, 1.28)	1.05 (0.92, 1.20)	1.05 (0.87, 1.28)	B				
**1.11 (1.02, 1.20)**	1.09 (0.93, 1.28)	1.09 (0.92, 1.30)	1.09 (0.97, 1.21)	1.08 (0.93, 1.26)	1.03 (0.89, 1.19)	J			
**1.21 (1.13, 1.29)**	**1.19 (1.03, 1.38)**	**1.19 (1.04, 1.36)**	**1.18 (1.10, 1.27)**	**1.18 (1.01, 1.38)**	1.12 (0.98, 1.28)	1.09 (0.98, 1.21)	H		
**1.27 (1.17, 1.39)**	**1.26 (1.07, 1.48)**	**1.26 (1.05, 1.50)**	**1.25 (1.12, 1.40)**	**1.25 (1.04, 1.49)**	**1.19 (1.10, 1.28)**	**1.15 (1.02, 1.30)**	1.06 (0.95, 1.18)	I	
**1.87 (1.62, 2.16)**	**1.85 (1.52, 2.25)**	**1.84 (1.49, 2.27)**	**1.83 (1.56, 2.15)**	**1.83 (1.48, 2.26)**	**1.74 (1.56, 1.93)**	**1.69 (1.43, 1.99)**	**1.55 (1.32, 1.81)**	**1.47 (1.30, 1.66)**	G

*Note.* Significant result is in bold. *Note.* A: acupuncture; B: electroacupuncture; C: acupoint application; D: moxibustion; E: acupoint catgut embedding; F: warm acupuncture; G: sham acupuncture; H: domperidone; I: itopride; J: mosapride.

**Table 8:** League table of SF-36.

B						
4.06 (−4.49, 12.61)	A					
5.12 (−8.48, 18.71)	1.06 (−9.52, 11.64)	E				
9.56 (−2.39, 21.51)	5.50 (−2.86, 13.87)	4.44 (−4.54, 13.42)	J			
**12.69 (3.94, 21.43)**	**8.63 (3.88, 13.37)**	7.57 (−4.02, 19.16)	3.12 (−6.49, 12.73)	I		
**13.95 (0.88, 27.03)**	9.89 (−0.01, 19.79)	8.83 (−0.87, 18.53)	4.39 (−6.36, 15.14)	1.27 (−9.71, 12.24)	H	
**17.18 (11.76, 22.61)**	**13.13 (5.24, 21.01)**	12.07 (−1.12, 25.25)	7.62 (−3.86, 19.11)	4.50 (−3.91, 12.91)	3.23 (−9.42, 15.88)	G

*Note.* Significant result is in bold. *Note.* A: acupuncture; B: electroacupuncture; E: acupoint catgut embedding; G: sham acupuncture; H: domperidone; I: itopride; J: mosapride.

**Table 9:** League table of NDLQI.

D					
2.68 (−6.72, 12.08)	A				
9.68 (−1.45, 20.82)	**7.00 (1.04, 12.97)**	B			
**13.33 (2.23, 24.43)**	**10.65 (4.74, 16.56)**	3.65 (−2.29, 9.59)	H		
**13.87 (2.61, 25.14)**	**11.19 (4.98, 17.41)**	4.19 (−1.71, 10.09)	0.54 (−6.81, 7.89)	I	
**19.90 (8.90, 30.91)**	**17.22 (11.50, 22.95)**	**10.22 (6.14, 14.30)**	**6.57 (0.09, 13.06)**	**6.03 (0.22, 11.84)**	G

*Note.* Significant result is in bold. *Note.* A: acupuncture; B: electroacupuncture; D: moxibustion; G: sham acupuncture; H: domperidone; I: itopride.

## Supplementary Materials

Supplementary Materials The supplementary materials include six parts: Part 1: checklist of the PRISMA extension for network meta-analysis. Part 2: forest plot of pairwise meta-analyses. Part 3: inconsistency tests based on the side-splitting approach. Part 4: inconsistency tests based on the loop-specific approach. Part 5: ranking probabilities, mean ranks, and SUCRA values. Part 6: comparison-adjusted funnel plots.
